# Important citation identification by exploiting content and section-wise in-text citation count

**DOI:** 10.1371/journal.pone.0228885

**Published:** 2020-03-05

**Authors:** Shahzad Nazir, Muhammad Asif, Shahbaz Ahmad, Faisal Bukhari, Muhammad Tanvir Afzal, Hanan Aljuaid

**Affiliations:** 1 Department of Computer Science, National Textile University, Faisalabad, Pakistan; 2 Punjab University College of Information Technology (PUCIT), University of the Punjab (PU), Lahore, Pakistan; 3 Department of Computer Science, Capital University of Science and Technology, Islamabad, Pakistan; 4 Department of Computer Science, College of Computer and Information Sciences, Princess Nourah bint Abdulrahman University (PNU), Riyadh, Saudi Arabia; KU Leuven, BELGIUM

## Abstract

A citation is deemed as a potential parameter to determine linkage between research articles. The parameter has extensively been employed to form multifarious academic aspects like calculating the impact factor of journals, h-Index of researchers, allocate different research grants, find the latest research trends, etc. The current state-of-the-art contends that all citations are not of equal importance. Based on this argument, the current trend in citation classification community categorizes citations into important and non-important reasons. The community has proposed different approaches to extract important citations such as citation count, context-based, metadata, and textual based approaches. The contemporary state-of-the-art in citation classification community ignores significantly potential features that can play a vital role in citation classification. This research presents a novel approach for binary citation classification by exploiting section-wise in-text citation frequencies, similarity score, and overall citation count-based features. The study also introduces machine learning algorithms based novel approach for assigning appropriate weights to the logical sections of research papers. The weights are allocated to the citations with respect to their sections. To perform the classification, we used three classification techniques, Support Vector Machine, Kernel Linear Regression, and Random Forest. The experiment was performed on two annotated benchmark datasets that contain 465 and 311 citation pairs of research articles respectively. The results revealed that the proposed approach attained an improved value of precision (i.e., 0.84 vs 0.72) from contemporary state-of-the-art approach.

## Introduction

Discoveries in science are always associated to the studies done by peers of the domain [1] This chain prolongs in the form of citations wherein prior state-of-the-art work is referred in some context. In scientific community, citation is deemed as an acknowledgment given to the cited research work. A citation is also considered as an indicator for analyzing different aspects like allocating Research funds [2], ranking the journals [3], ranking the researchers [4], finding the research topics, finding the latest research trends, ranking of institutions, ranking of countries [5].

In scientific community, there has been a continuous debate that pertains to the degree of importance among multifarious citation reasons [6–8]. There could be various reasons behind citing a particular study such as the utilization of previous knowledge, enhancing the results, comparison of results, etc. Most of the citation analysis community grants equal weight to all the citation reasons. However, similar to [6, 9], we believe that citation quality should not be assessed solely based on its count rather quantitative and qualitative approaches should be accommodated for effective citation analysis. The scientific community suggested that citation reasons should also be given weightage instead of utilizing only the citation count of research articles [10]. The researchers are of the view that a citation presenting the background knowledge and a citation made in context of results comparisons, can never be of the same significance and importance. In most of the research articles, 40% of citations are made for depicting the background knowledge or for general acknowledgments [8]. The research community has turned its focus towards finding the important citations for calculating the research achievements. A citation is considered important if the work is an extension or is inspired by previous work. On the other hand, a citation is considered non-important if it is done for criticizing or presenting the background knowledge.

The researchers have presented different models and techniques for classification of citations into different categories. Gradually, the classification trend transformed from manually asking the authors about important citations to automatically classification of citations [11]. Many researchers started to automatically classify the citations into different groups and introduced lexical matching rules. The reasons were the basis for the classification of citations. Gradually the different classification groups were merged into two groups [10, 12, 13], and this was termed as binary classification. The current approaches are focusing on content [6] or meta-data [13] of research articles to find out the important and non-important citations such as (1) Content-based Approach, (2) Citation count, (3) Citation Context Approach, (4) meta-data based approach, etc. In content-based, context-based and meta-data based approaches, researchers tend to find the similarities among the text corpora [12]. If the textual similarity is high, the citation is considered as important. While on the other hand, in the citation count-based approach, the high frequency of a citation is considered as important [14]. The results produced by the state-of-the-art work, are insufficient for making potential decisions. Therefore, there is need to improve the results.

This research introduces a novel technique for identifying the important citations [15] of research articles. To perform this experiment, we utilized two benchmark datasets, first dataset is collected by Valenzuela and annotated by two domain experts [12], and another was collected by Faiza et al. [13] which was annotated by the actual authors of the papers from Capital University of Science and Technology, Islamabad (CUST). This study focuses on three different features like citation count, similarity score and section-wise weights for in-text citations. For citation count feature, all direct or indirect citations were counted. For the similarity score, we calculated cosine similarity among citing and cited research articles and the section-wise citation frequency is computed for each given pair by using an automated approach. Multiple Regression and Neural Network are employed for appropriate weight calculation. The obtained weights were further multiplied to the section-wise citation frequencies. For evaluation, performance measures such as precision, recall, and f-measure are considered. To classify the citations on the basis of above-stated features, we used three machine learning algorithms: (1) Kernel Logistic Regression, (2) Support Vector Machine, and (3) Random Forest.

The results with Neural Network were observed healthier, therefore, Neural Network was designated for identification of important citations. The outcomes of the study are determined on the basis of a comparison of the proposed approach with contemporary state-of-the-art approach [13]. The comparison revealed that proposed model attained improved value of Precision, (i.e., from 0.72 to 0.85).

## Related work

Citation serves as an acknowledgment to the previously done work by researchers. Citation analysis is used for exploring and calculating different aspects of research, such as the ranking of the researchers, impact factor calculation of the different journals, ranking the countries, ranking of institutions, allocation of research funds, allocating awards, making policies, etc. Citations were first analyzed by Garfield [14], and the author investigated the correlation between the awards and the number of citations. This idea was further adopted by Inhaber [2], and he tried to find the relation of research fund allocation of awards with citations. Moravcsik et al. [8] developed a technique for the classification of citations into different categories and reported that citations depend on different reasons therefore, they cannot be considered equally important. The dataset used by the researcher consisted of 30 research articles with 720 citations. The researcher found that 14% of citations were negative. After this research work, the research community proposed different techniques to identify the reasons for the citations. The approach was further adopted by Rosing [16], and the author classified the citations into 13 different groups with respect to their reasons. Initially, authors were manually interviewed about the reason for citations. However, it becomes impractical for large number of documents to manually specify the reasons. Therefore, the research community turned its focus towards the automation of citation classification. To automatically classify the citations into different categories with respect to their reasons, Finney [11] proposed a first semi-automated approach to classify citations. This research was served as a base for the research community. In her work, the author used a citation function considering the citation location and the cue words as well. She classified the citation into seven different groups. Giles et al. [17] was the first who introduced the automatic citation indexing engine that was named as CiteSeer. It is a search engine and digital library specifically containing literature from the field Computer Science.

The automatic classification of citations was further enhanced by Garzone [18]. The author introduced a citation classification technique that was fully automated. The author pointed out deficiencies in the work of Finney and covered those deficiencies by classifying the citations into 35 different categories. The author introduced 195 lexical rules with 14 rules for parsing the documents. To perform the experiment a dataset containing 20 different research articles was utilized. The approach performed well with known research papers, but for the unknown dataset, the approach showed average results. There was also another limitation of work that there were many classes for citations, and it was difficult to classify the citations. A system named KAFTAN was presented by Pham et al. [9]. This system classified the citations into four different categories considering the cue word phrases based on aspects like (1) fundamental, (2) supporting, (3) limitation and (4) comparison. A comprehensive approach for citation index was introduced by Henry et al. [19]. The proposed approach was able to consider the direct and indirect citations for finding the influence of research papers. The author reported that CCI showed accurate results with respect to the research articles. The author collected the citations from the site http://scholar.google.com, which is a dataset of citations consisting of more than 288,404 records. The author claimed that the CCI method performed better than the state-of-the-art SCI and PageRank used for the identification of research articles. The techniques which have been discussed until now are content-based. The research community has also tried to focus on the behavior of a citation [3]. In citations analysis, all the approaches consider the citations with equal importance. However, the researchers are of the view that each citation done by a researcher serves different reasons. Therefore, it would not be effective to treat all the citations on equal basis. If we describe important and non-important citations, it would enhance the quality of citation analysis.

A supervised machine learning approach was proposed by Teufel [20] to automate the classification of citations. This classification was done considering the linguistics features. The citations were classified into four different categories and 11 subcategories. To tag the citations, the author used a manual approach but other features such as self-citations were detected automatically. The machine learning algorithm was used, and for the validation of results, 10-fold technique was utilized. The reported accuracy by the author was 0.80, while the F-measure was 0.71. After that, a technique was proposed by Sugiyama et al. [21] for the classification of citations into two categories, such as citing and non-citing. SVM was used citation classification, and different features were incorporated, such as sentences, positions, and nouns. The author revealed that for training, context and proper nouns were more appropriate. The same machine learning algorithm SVM was used by the Agarwal [22], who classified citations into eight different categories. The author used a dataset of 43 research articles. The score of F-measure was found as 0.76. Dong et al. [23] performed the classification of citations into three categories, such as (1) Negative, (2) Positive and (3) Neutral as well.

Further citation classification was performed by Jochim et al. [24]. The author tried to find citations that have more effect on research work as compared to the other citations. To perform the experiment, lexical features were used that classify the citations considering the lexicons of context. The dataset for the research work was collected from the site of ACL Anthology. A keyword-based technique for the classification of citations into positive and negative classes was introduced by Kumar [25]. The sentences were extracted from ACL Anthology. The author performed sentiment analysis and classified the citations into positive and negative classes. Zhu et al. [10] was the pioneer of the concept of classifying citations into two classes, which is termed as binary citation classification. For binary classification, the author introduced two terms such as influential and non-influential citations.

The terms influential and non-influential were refined by Valenzuela [12]. The author named the terms as important and non-important for citations. A dataset of 465 citation pairs was used, which was extracted from ACL Anthology. The dataset was further annotated by two domain experts, they classified the citations into important and non-important classes. Both the experts were 93.9% satisfied with the annotation. For the classification of citations author used 12 different features such as direct citations, indirect citations, the similarity of abstracts, citation count, etc. For training on features, two algorithms, SVM and Random forest were utilized. The produced results were in the form of performance measures and were reported as precision 0.65, and the value of recall was 0.90. Faiza et al. [13] further extended the research of binary classification of citations. Two datasets were used for the experiment. The author first utilized the annotated dataset of Valenzuela and the second dataset was collected having 324 paper-citation pairs which were manually annotated by the actual citing authors from department of Computer Science at CUST. This experiment was conducted utilizing the Meta-data of different research articles. The author used eight different features such as keywords, similarity, and dissimilarity of title, the similarity of the abstracts, etc. According to the author best results were achieved by the Random Forest algorithm. The maximum value of precision was increased from 0.65 to 0.72, but the value of recall was decreased from 0.90 to 0.70 as well. On the basis of these values of precision and recall, the potential decisions are difficult to make therefore the need of the hour is to improve the results.

## Methodology

**Fig 1:**
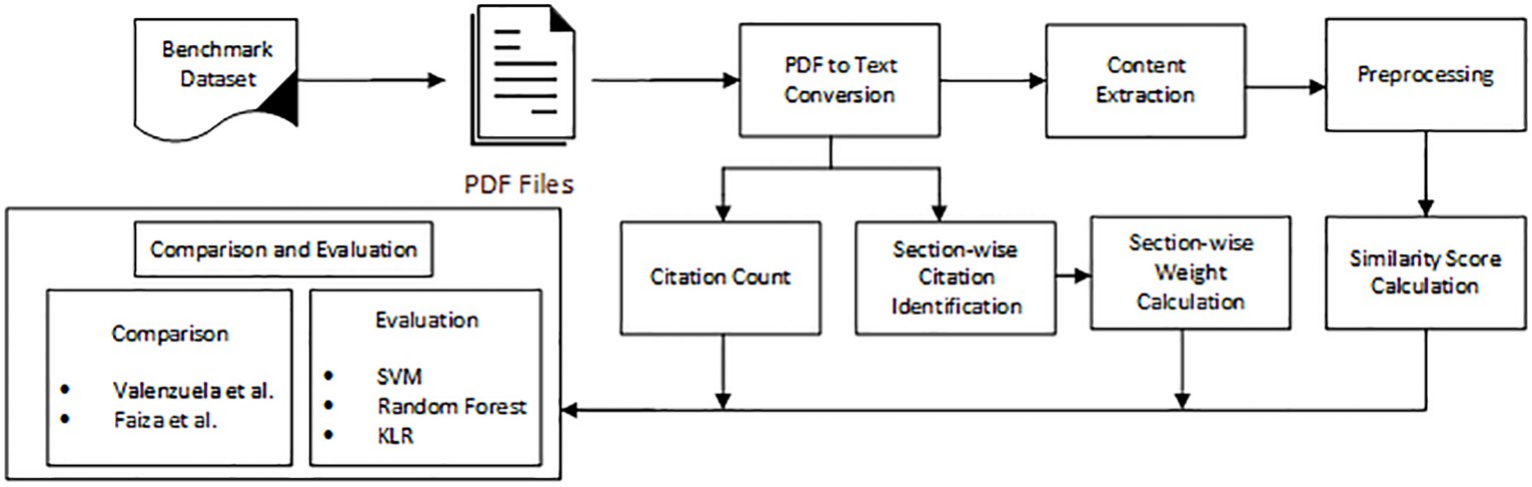
Overall methodology diagram.

This research is conducted for the classification of citations into important and non-important classes. The overall proposed methodology is presented in Fig 1. The key modules of the methodology are Data collection and PDF to Text conversion, citation count calculation, similarity calculation of citation pairs, and section-wise weight calculation. The classification of citations was made using machine learning algorithms. The results were produced considering the performance measures such as precision, recall, and f-measures. The detail of these modules is provided in subsequent subsections.

### 0.1 Data collection

To conduct this research, we used an openly available data set, One of them was collected by Valenzuela et al. [12] and annotated by two domain experts and another one was collected by Faiza et al. [13] which was annotated by the actual citing authors. We utilized this data set because of its standard nature as this data set was used in well-known research, which was published in the reputed journal. The data set was collected by Valenzuela, contains 20,527 research articles. This data set was extracted from ACL Anthology [26] with the citation graph. The total numbers of citations were 106,509, as it was difficult to label all the citations. Therefore only 465 citation pairs were labeled by the domain experts that belonged to the computer science field. The dataset is available at https://allenai.org/data/data-all.html. The annotators created the four different groups from 0 to 3, considering the importance of the citations, 0 representing relevant work, 1 representing the comparison, 2 indicating utilizing the work, and 3 for indicating extending the work done. These four groups were further merged into two categories, such as 0 and 1 categories were merged and labeled as 0 indicating non-important work while the remaining two categories 2 and 3 were merged into a single category labeled as 1 indicating important work.

As shown in Table 1, there are 85.4% citations that are non-important, and 14.6% citations which are non-important. A collection of 465 paper-citation pair was utilized by Valenzuela et al. and Faiza et al. [13]. The articles were scraped using their Id’s and by embedding those Ids at the end of the link http://www.aclweb.org/anthology/. After the scraping, we investigated that some Ids were missing, such as 1) W07-2058, 2) L08-1584, 3) L08-1328, and 4) L08-1267. On the other hand, four articles were unable to be scraped because of their unavailability. We performed our research on the dataset containing 457 research articles.

**Table 1:** Statistical information of data set D1.

Classes	Annotation		Label	Citations
0	Related Work	Non-important	0	398
1	Comparing with work	Non-important		
2	Using work	Important	1	67
3	Extending the work	Important		

The first column in Table 2, depicts the annotator, the second column consists of the ids of cited papers, and the next column is for ids of citing papers. In Follow-up column, 0 represents non-important, and 1 represents important citation.

**Table 2:** Annotated data set D1.

Annotator	Paper	Cited-by	Follow-up
A	H05-1079	W08-2004	0
A	H05-1079	N06-1005	1
A	I05-2038	C10-1076	0
B	P05-1045	D11-1135	0
B	P05-1045	D11-1141	1

As the numbers of citation pairs are not sufficient for analysis, therefore, another dataset D2 was also utilized, which was previously used by Faiza et al. This dataset originally consists of 311 citation pairs with titles and their ids. The research articles were from different publishing sites such as Elsevier, IEEE, Sciencedirect, etc. The citations were annotated by actual citing authors. These authors are from different fields of computer science such as networking, information retrieval, semantic analysis, image processing, etc.

In Table 3, the first column is describing the four classes of citations that were further merged into two classes. The label 0 depicts non-important citations and 1 depicts important citations. The total 216 citations were annotated as non-important, and 95 citations were annotated as important. The following Table 4, presents the Id’s and titles of data set D2.

**Table 3:** Statistical information of data set D2.

Classes	Annotation		Label	Citations
0	Related Work	Non-important	0	216
1	Comparing with work	Non-important		
2	Using work	Important	1	95
3	Extending the work	Important		

**Table 4:** Titles and Id’s of data set D2.

ID	Titles
1	JavaSymphony: A Programming and Execution Environment
2	Scheduling JavaSymphony Applications on Many-Core Parallel Computers
3	On the Evaluation of JavaSymphony for Heterogeneous Multi-core Clusters
4	Parallelism as a Concern in Java through Fork-join Synchronization Patterns
5	The JavaSymphony Extensions for Parallel GPU Computing

The first column in Table 4, is consisted of the Id’s of research articles and the second column contains their titles. Utilizing the titles, research articles can be extracted from different journals. The total number of research articles are 324 that further creates 311 citation pairs. Out of 311 citation pairs, 69.45% are important and 31% are non-important.

As presented in Table 5, each row depicts citation pair such as in first column, there are id’s of research articles that were cited, and second column is consisted of the id’s of citing research articles. While the last column contains annotation of citation pairs, where 0 represents the non-important citations, and 1 represents important citations. These citation pairs were annotated by the actual authors of the research articles from Capital University of Science and Technology, Islamabad. This data set is available at https://www.kaggle.com/shahzadnazir93/annotated-citation-pairs.

**Table 5:** Annotated data set D2.

PaperID	CitedBy	Fine Grained Value
1	2	1
1	3	1
1	4	0
1	5	0
1	6	0

### 0.2 PDF to Text conversion

The manipulation of PDF files is very complex and difficult. On the other hand, text files are easy to parse. Therefore, we focused on converting PDF files to Text files. To carry out the conversion we used an openly available tool named XPDF [27]. It can convert the PDF files to Text files. It is available on the Github site and designed in the R language. This tool is efficient for conversion; therefore, we converted all the PDF files into text files using this tool.

### 0.3 Citation count

This phase of the experiment is related to the frequency calculation of citations. It is an attempt to discover the number of occurrences of citations in research articles. If paper A is cited three times in citing paper, the frequency of the citation would be 3. In this step, the frequencies of cited papers were calculated from citing papers. The citation occurring in any section is given equal importance. To calculate this citation count, ParCit [28] a publicly available tool, was utilized. It extracted the citations from research articles by considering their structures.

### 0.4 Similarity score

The similarity score between citing and cited articles could be considered as a potential feature to determine importance of a citation. For similarity score calculation, we used the whole content of research articles. To calculate the similarity score among each pair, we first extracted the text from text files, after that we performed pre-processing to remove the stop words and converted the words into their base form with stemming. We further detected the potential key terms using TF-IDF and for similarity calculation we implemented the cosine similarity algorithm on extracted key terms. Cosine similarity algorithm considers the semantics of content and calculates the score value.

### 0.5 Section-wise in-text citation identification

The calculation of section-wise weights consists of four further sub-modules such as (1) Parsing Text files, (2) finding the location of citations, (3) calculating the section-wise citation frequency, and (4) weight assignment to sections. This step includes fetching the citation from each section of the research article and then calculating the frequencies of citations with respect to section. To perform this experiment, we considered four sections [15], which are common in almost all research articles. The sections are as follows:

- Introduction
- Literature Review
- Methodology
- Results and Discussions

#### 0.5.1 Parsing Text files

To extract the citations from text corpus, we need to parse the text files. Therefore, for parsing the text files, we used ParCit [28], which is also an openly available tool. This is most common that efficiently parses the text files. It is a Conditional Random Field Model, and we do not need to train it as it is already trained. It tokenizes the sentences and labels those tokens. It has the ability to identify the reference strings from the corpora and locate the citations in context. ParCit outputs the different elements of research articles such as (1) Citations, (2) Abstracts, (3) Sections, (4) keywords, (5) Title and (6) Authors. It parses the logical structure of the text files as well, and the bibliographic portion is automatically extracted.

#### 0.5.2 Finding location of citations

To locate positions of a citation was a complex task, but because of the same pattern of all the citations, the process became quite feasible. First, the sections were extracted, and in each section, the program located the citations. The data set is consisted of two categories of research articles the “Cited papers” and the “Citing papers.” The Author’s name and year of publishing were the major factors for locating the citations. As the citations start and end with a small opening and closing brackets, the name of the author and the publishing year were matched with the author’s name and publishing year of the cited paper. This pattern was matched in sections for locating citations, and the citations were efficiently located.

#### 0.5.3 Calculating the section-wise citation frequency

The frequency of citations is related to the number of their occurrence in the text corpora. This is a simple and quantitative approach where we count the occurrence of citations. If a paper is cited 5 times, the frequency of the paper would be 5. While counting the citations, we considered the sections where the in-text citation was made. To conduct the research, we focused on the most common sections of research articles where researchers do the citations. The focused sections were as 1) Introduction, 2) Literature, 3) Methodology, 4) Results and Discussions. For these sections in research articles, the citations were counted. Table 6 shows the sections and number of citations in those sections, such as the first row shows that a specific paper is only cited once in the Introduction section of the citing paper. Similarly, the rest of the rows are indicating citations of a paper in different Sections.

**Table 6:** Section-wise citation frequency.

Annotator	Paper	Cited-by	Follow-up	Total Frequency	Introduction Frequency	Literature Frequency	Methodology Frequency	Results Frequency
A	H05-1079	W08-2004	0	1	1	0	0	0
A	H05-1079	N06-1005	1	4	4	0	0	0
A	I05-2038	C10-1076	0	1	0	0	1	0
B	P05-1045	D11-1135	0	1	0	0	0	1
B	P05-1045	D11-1141	1	2	0	0	1	1

#### 0.5.4 Weight assignment to sections

To find out the appropriate weights for sections, we used Multiple Regression and Neural Network that are supervised machine learning models. These two supervised models have been utilized by different researchers for calculating the weights. Karakaya et al. [29] calculated the relative weights of independent variables with respect other variables using Multiple Linear Regression, to find out its robustness. The higher weight variables were termed as important. Multiple Regression [30] considers two or more independent or explanatory features and a single dependent or response feature. While Choi et al. [31] analyzed the susceptibility of landslide, by calculating the weights for each area with Neural Network. Therefore, to perform the experiment, Linear Regression and Neural Network was focused. In this experiment, the independent features are the different sections of the research article, and the dependent one is the Follow-up feature. The main idea behind this is to predict the dependent feature based on independent features. This model fits the linear equation to the training data. Each value of independent feature X has an influence on predicted Y. The equation of Multiple Regression is as follows:

$$Y=\beta_{0}+\beta_{1}X_{1}+\beta_{2}X_{2}\ldots \ldots +\beta_{k}X_{k}$$ (1)

*β*_1_, *β*_2_… *β*_*k*_ are the coefficients and X1, X2,… Xk are the independent features that would be multiplied with coefficients. *β*_0_ is the y-intercept or a constant which has to be added. This model estimates values of coefficients which in our case would be the weights. To optimize the weights, model uses cost function that minimizes the error between actual and predicted values. The cost function used for Multiple Linear Regression is as follows:

$$MSE=\frac{1}{2N}\sum (y_{i}-{\left(\beta_{1}X_{1}+\beta_{2}X_{2}\ldots +\beta_{k}X_{k}\right)}^{2}$$ (2)

While the Neural Network consists of neurons, which receive certain inputs and those inputs are further multiplied with weights. A neuron is a function that collects the information and classifies it considering the structure of the Neural Network. Sigmoid Function was used as an activation function that can be expressed as follows:

$$\sigma (z)=\frac{1}{1+e^{-z}}$$ (3)

Multiple Regression and Neural Network models were trained on 60% of data values and tested on the rest of the 40%. The section-wise frequency values of citations were provided as independent features and follow up as a dependent feature. The best-suited weight values on training data were considered as appropriate weights. These optimized weight values were used for testing the data to obtain the results.

## Results and discussion

As explained earlier, two datasets D1 and D2 were used to perform experiments. The first dataset D1 was collected by Valenzuela, and the second dataset D2 was collected by Faiza et al. In Dataset D1, there were 457 citation pairs that were utilized for finding the important and non-important citations. The statistics of dataset D1 are explained in the following Table 7.

**Table 7:** Statistics of dataset D1.

Citation Pairs	Important	Non-important	Sections	Citations
457	69	388	Introduction	155
			Literature Review	131
			Methodology	404
			Results and Discussions	77

In the above-given table, we have different sections and their citations, respectively. In dataset D1, out of 457 citation pairs, 69 were found annotated as important and 388 as non-important. In the Introduction sections, total citations were 155, in Literature Review contained 131, Methodology contained 404, and 77 citations were extracted from the Results and Discussion sections. The maximum citations were found in Methodology sections while minimum citations were 77 which were found in the Results and Discussions section. As far as the dataset D2 is concerned, we received this dataset from the citing authors. D2 contains 311 pairs. For Ids 32, 71, 135, 152, 156, 157, 163, 164, 175, 180, 187, 191, 192, 195, 198, 199,216, 222, 228, 230, 235, 244, 246, 262, 266, 290, 303, 316 and 317, we were unable to find the references, citations to download the research articles. Therefore the further research was conducted on 282 citation pairs of D2. The statistics of D2 are explained in Table 8 which is given below.

**Table 8:** Statistics of dataset D2.

Citation Pairs	Important	Non-important	Sections	Citations
282	89	193	Introduction	157
			Literature Review	122
			Methodology	116
			Results and Discussions	69

In the D2 dataset, the total numbers of tuples were 282, where 89 were important, and 193 were non-important. The citations in the introduction sections were 157, citations in Literature Review were 122, in Methodology 116, and in Results and Discussions, 69 citations were found. Here the maximum citations were 157, which were found in Introduction sections, and minimum citation count was 69, found in Results and Discussion sections.

We utilized three different features, such as citation count of research articles, similarity measure, and the third feature was the key feature, which was based on assigning the appropriate weights to the different sections where the citations are commonly made by considering the in-text section-wise citation count. As far as citation count is concerned, we considered all the citations for each citation pairs such as direct citations and indirect citations. To find out the similarity feature, we first extracted the potential key terms from the text corpus of research articles of citation pairs.

After that, we applied cosine similarity on the extracted key terms and calculated the similarity score of research articles. S1 and S2 Files contain all the utilized features for Datasets D1 and D2. For calculating weights, we first extracted section-wise citations and then applied Multiple Regression and Neural Network on them. The weights were further multiplied with section-wise in-text citation counts. The resultants of the multiplications were used as a feature for important citation identification. For classification of citations, we used three machine learning algorithms (1) Support Vector Machine (SVM), (2) Random Forest (RF), and (3) Kernel Logistic Regressions (KLR). To solve the problem of imbalance class, we used the SMOTE filter that virtually produces the instances by considering the nearest neighbors. This filter is used to balance the classes if the one has a lower number of instances or a larger number of instances. Further 10 fold cross-validation was performed on the dataset. In 10 fold cross-validation, 9 parts of data are used for training, and 1 part of data is used for the testing. The results are explained in the form of performance measures such as precision, recall, and f-measure. These are explained in the following subsections.

### 0.6 Performance measures for section-wise citation weights

To find out appropriate weights for sections, we utilized machine learning algorithms. For weight calculation, we utilized machine learning algorithms that are Neural Network and Linear Regression. As there was more than one section, therefore Linear Regression was termed as Multiple Regression. After applying Multiple regression, we obtained the weights, and to make the accumulative sum of weights as 1, and we further normalized the weights of the sections are given in Table 9.

**Table 9:** Appropriate normalized weights by Multiple Regression.

Sections	Weights	Weight Rank
Introduction	0.1891921316	3
Literature Review	0.1470393226	4
Methodology	0.3663496373	1
Results and Discussions	0.2974189085	2

The optimal obtained weights by the Multiple Regression (MR) are presented in Table 9. The value of Y-intercept was kept 0 so that there is no need to add the constant value of y-intercept in the calculation. Similarly, we applied the Neural Network and obtained the weights. The weights were further normalized as presented in Table 10.

**Table 10:** Appropriate normalized weights by Neural Network.

Sections	Weights	Weight Rank
Introduction	0.19095378	3
Literature Review	0.17626289	4
Methodology	0.28763501	2
Results and Discussions	0.34514832	1

It can be observed that Multiple Regression assigned a maximum weight to Methodology; on the other hand, Neural Network assigned a maximum weight to the Results and Discussion section that is more logical. For each citation pair, these weights were multiplied with existing citations in different sections. Tables 11 and 12 illustrate the multiplication of in-text section-wise citation count with weights produced by Multiple Regression and Neural Network respectively.

**Table 11:** Multiple Regression results on section-wise citation count.

Total Frequency	Introduction	Literature Review	Methodology	Results
2	0	0	0.7326992746	0
1	0.1891921316	0	0	0
2	0.1891921316	0.1470393226	0	0
1	0	0	0	0.2974189085
1	0	0	0	0.2974189085

**Table 12:** Neural Network results on section-wise citation count.

Total Frequency	Introduction	Literature Review	Methodology	Results
2	0	0	0.57527002	0
1	0.19095378	0	0	0
2	0.19095378	0.17626289	0	0
1	0	0	0	0.34514832
1	0	0	0	0.34514832

As shown in Tables 11 and 12, in the first row, the total frequency is 2, and both times, the citation was made in the methodology section. Therefore, the weight of this section was multiplied with the frequency of citation in the methodology section. In the second row, the research article is cited only once in the Introduction section. Therefore 1 value in the introduction was multiplied with the weight of the section. After that, we classified the citations on the basis of these in-text citations weigh values. This was the key feature to conduct the research work. The three algorithms, such as support vector machine, kernel logistic regression, and random forest, were applied to this feature, all the algorithms produced different results of performance measures on dataset D1 and D2 which are presented in the form of precision, recall, and f-measure.

**Fig 2:**
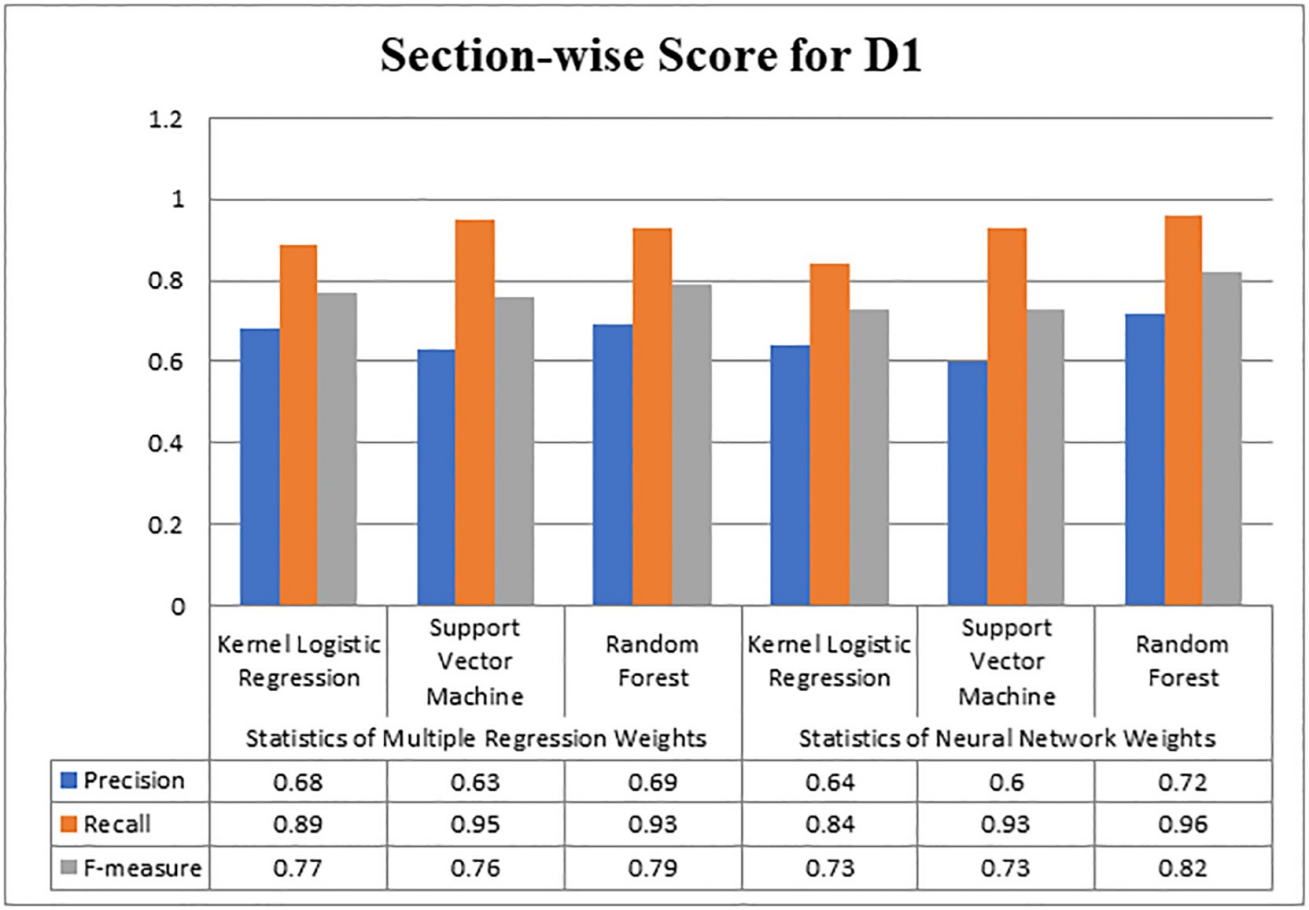
Evaluating section-wise weight score for D1.

In this experiment, initially, we considered only one feature, which was section-wise citation weight. By considering this feature, we further classified the citations into two classes important and non-important ones. The maximum attained value of precision is 0.72, produced by the Random Forest algorithm as presented in Fig 2. The maximum value of recall 0.96 was gained by the Random Forest, and the maximum F-measure was again achieved by the Random Forest that was 0.82. Overall for dataset D1 Random Forest produced better results than other classification algorithms.

**Fig 3:**
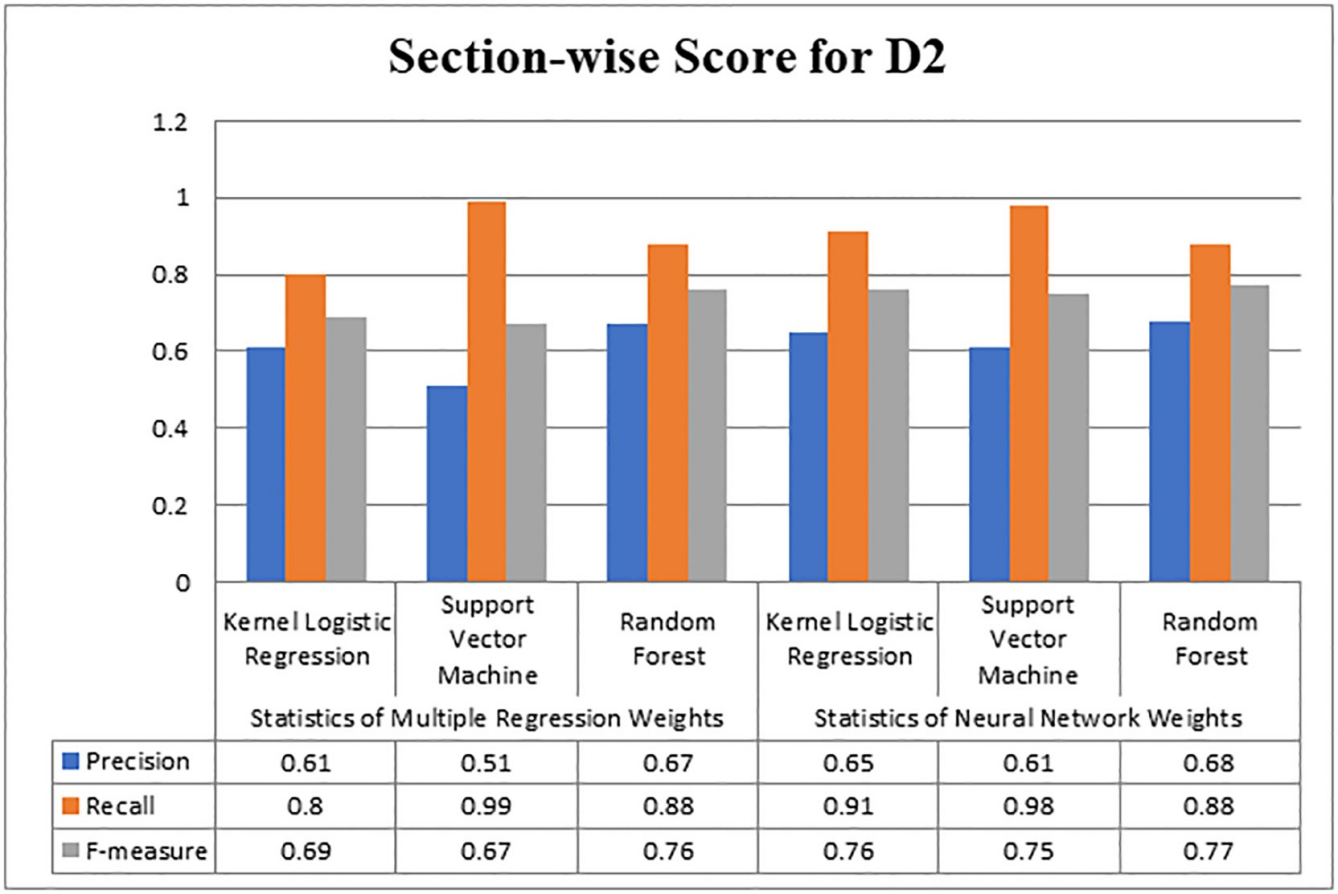
Evaluating section-wise weight score for D2.

Fig 3 illustrates the results of performance measures gained by the three classifiers on dataset D2. It can be observed from the figure that the maximum precision value was obtained by the Random Forest algorithm which is 0.68. From Figs 2 and 3, it becomes obvious that maximum precision was produced by the Random Forest on weights that were obtained by the Neural Network.

### 0.7 Combined results

The approach of Faiza et al. achieved a maximum precision of 0.72 which was an extension in Valenzuela’s work that gained 0.65 precision. We further improved these results and gained precision of 0.85 with Random Forest on the same dataset that was used by Valenzuela and Faiza et al. These results were produced by combining the features such as (1) Content Similarity, (2) Citation Count and (3) Section-wise In-text Citation Weights. Among all these features, the new feature that was introduced in this research was the Section-wise In-text Citation Weights. The results of these features have been discussed earlier. In this subsection, we present results with the combined effect of all three features.

**Fig 4:**
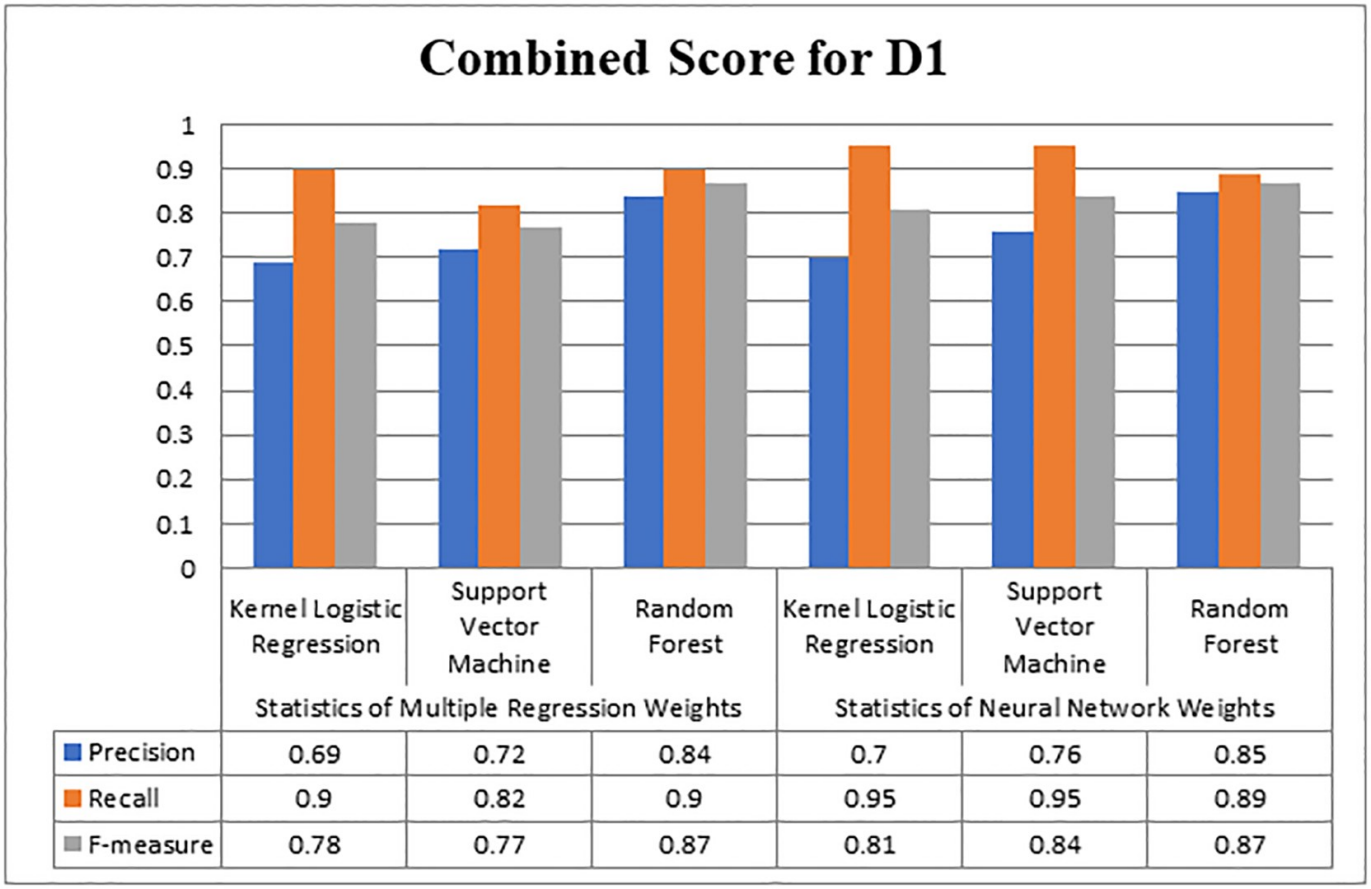
Overall score for D1.

As shown in Fig 4, with Multiple Regression weights, the maximum precision was attained by Random Forest 0.84, Support Vector Machine gained 0.72, and Kernel Logistic Regression produced 0.69 precision. While the value of recall by RF was 0.90, by Support Vector Machine 0.82 and by Kernel Logistic Regression, it was 0.90. The maximum F-measure was achieved 0.87 by Random Forest. On the other hand, with Neural Network weights, maximum precision was 0.85, which is slightly higher than the maximum precision value gained using Multiple Regression weights. While Kernel Logistic Regression and Support Vector Machine produced 0.70 and 0.76 precision, respectively. As far as the recall is concerned, the maximum recall value was 0.95 produced by KLR and SVM. The value of f-measure was maximum with Random Forest 0.87. KLR and SVM produced 0.81 and 0.84 values of f-measure with Neural Network weights. Therefore can be inferred that the Random Forest performed well on D1.

**Fig 5:**
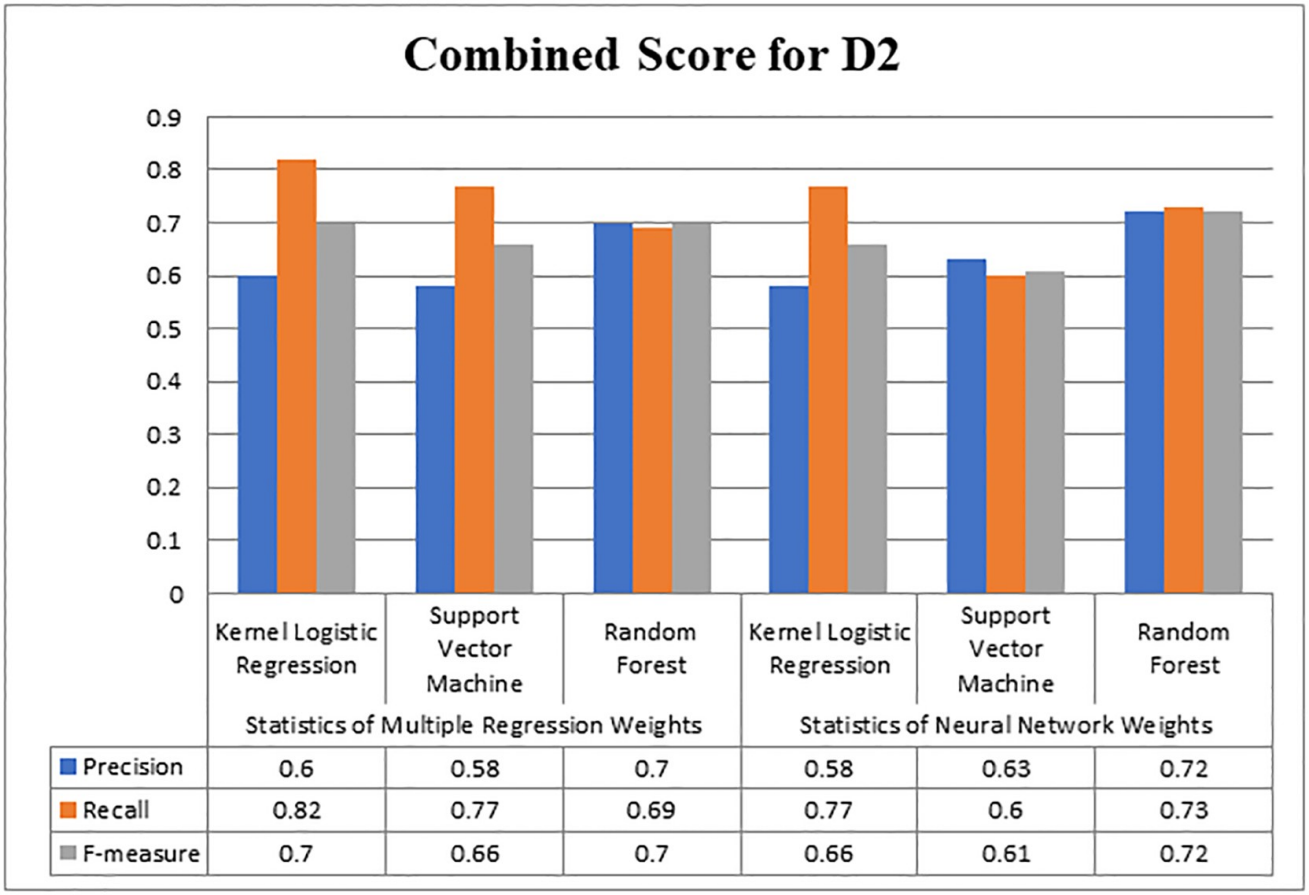
Overall score for D2.

Fig 5 graphically represents the scores of Kernel Logistic Regression, Support Vector Machine, and Random Forest. The maximum score of precision on D2 was 0.72 with Random Forest, while Support Vector Machine produced 0.63, and Kernel Logistic Regression produced 0.60 precision values. As far as the value of recall is concerned, Kernel Logistic Regression, Support Vector Machine and Random Forest attained 0.82, 0.77, and 0.73, respectively. Kernel Logistic Regression achieved a maximum 0.7 value of f-measure, while Support Vector Machine and Random Forest gained 0.66 and 0.72 value of f-measure respectively. Random Forest performed better in terms of precision and f-measure. Overall, the results produced with Neural Network as compared to Multiple Regression were more acceptable. Neural Network outperformed on both datasets, as it offers the back-tracking and weight adjustment at each neuron. Therefore, for finding the important citations, weights with Neural Network would be considered.

### 0.8 Comparison

**Fig 6:**
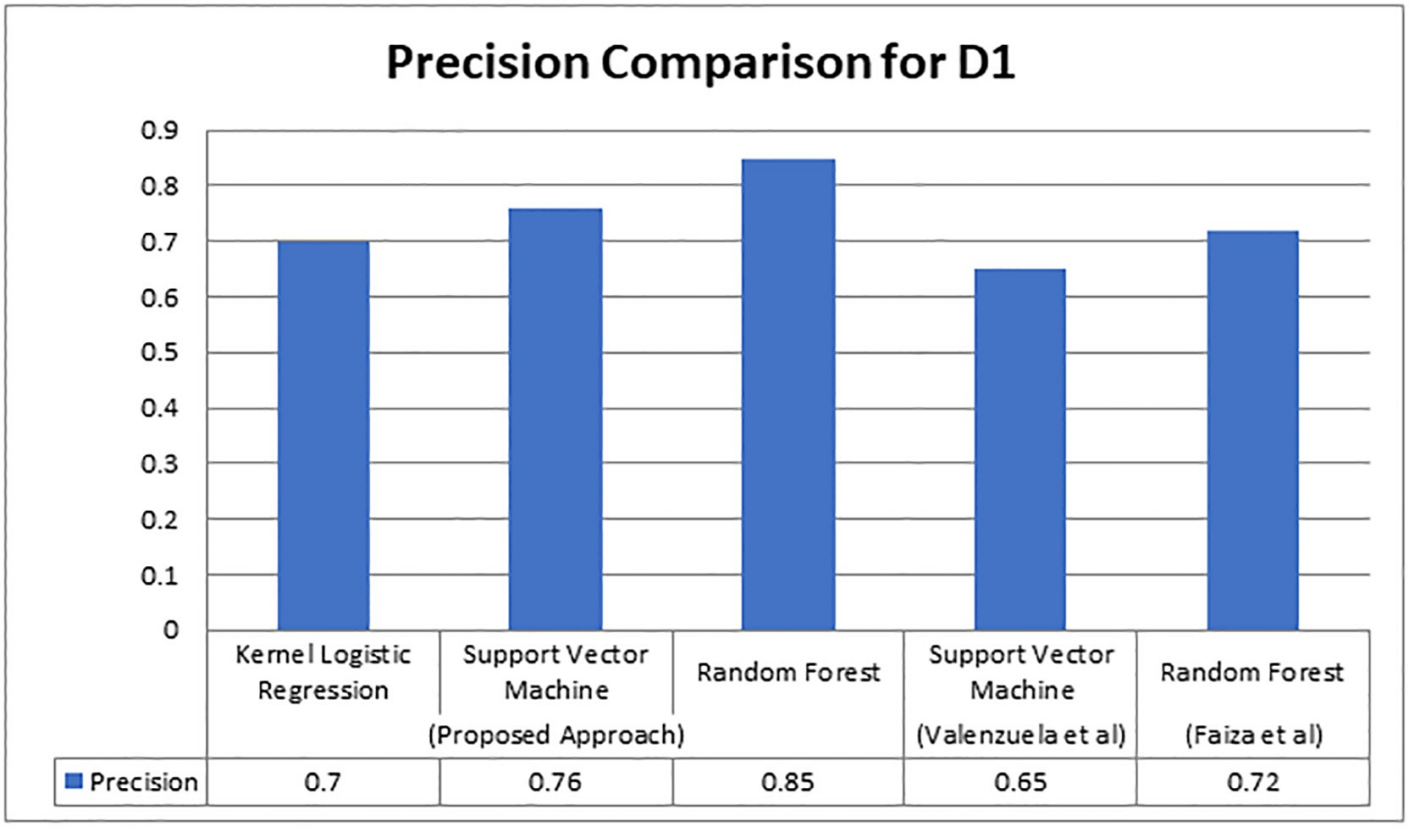
Results comparison of Valenzuela’s dataset.

The study presented by Valenzuela et al. [12], utilized twelve features and gained 0.68 precision, and the features of Valenzuela’s approach were content-based and metadata-based. While state-of-the-art approach considered only metadata and produced better precision such as 0.72 with Random Forest classifier. We utilized the same dataset, which was collected by Valenzuela, and applied our proposed technique, and attained improved results. The feature that boosted the results was mainly section-wise in-text citation weight. We achieved a precision of 0.85 which is significantly higher than state-of-the-art work. The graphical representation of the comparison is given in the following Fig 6.

**Fig 7:**
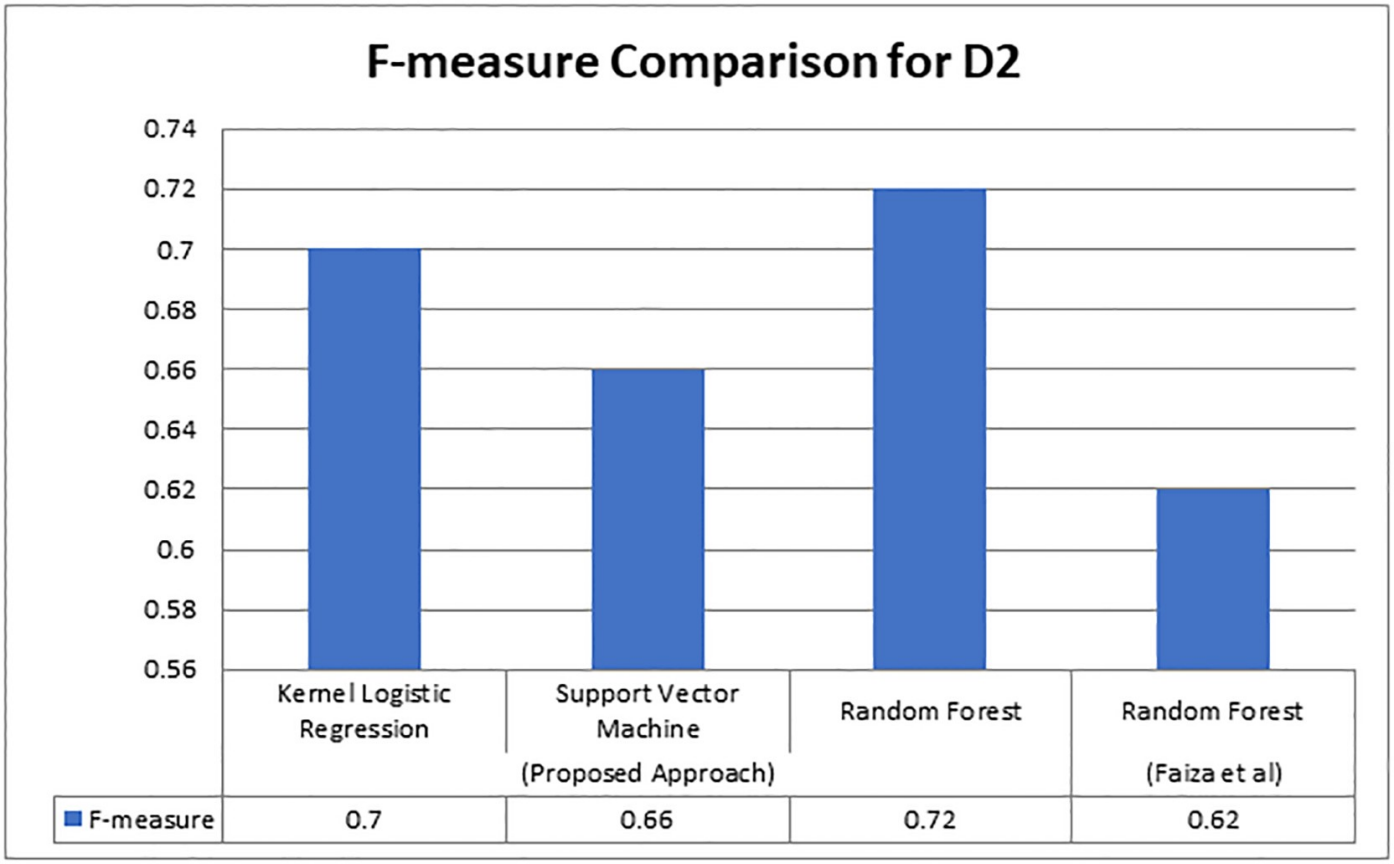
F-measure comparison of dataset D2.

As presented in Fig 6, we can observe that our proposed technique produced better results on the same dataset where Valenzuela achieved 0.65, Faiza achieved 0.72, and our proposed technique achieved 0.85. For dataset D2 that was utilized by Faiza et al. [13], we compared the value F-measure, it is graphically represented in Fig 7 as follows.

The above Fig 7 is a graphical comparison of F-measure values gained on Dataset D2. As Faiza et al. [13] achieved F-measure of 0.62, while our proposed system achieved 0.72 score of F-measure with Random Forest, 0.66 with Support Vector Machine, and 0.70 with Kernel Logistic Regression. We claim that the proposed methodology is quite feasible than the compared contemporary approaches, therefore, this study is a significant contribution in citation classification community.

## Conclusion

In scientific community, a citation could be considered as an important indicator to provide feedback regarding particular study. Normally, when an author cites someone’s work, he/she cites that work as an acknowledgment. Citations play important role in calculating the impact factor of journals, ranking the researchers, ranking of countries, allocating research funds, ranking of institutions, etc. While measuring these research aspects, citations are treated equally. The research community claims that all the citations do not have the same importance, which could raise doubt on pure citation-count based approaches. To identify important citations different approaches have been proposed, such as context-based, Metadata-based, etc. However, these approaches do not consider some of the potential parameters for classifying citations. We conducted this study for identifying important citations using three different features (1) assigning the appropriate weights to sections, (2) calculating direct and indirect citations, and (3) measuring the similarity of research articles. To perform the experiment, we used two datasets, collected by Valenzuela et al. and Faiza et al. The main feature introduced in this study is section-wise in-text citation weights. We assigned appropriate weights to logical sections of research papers where in-text citations are found. To assign the weights to sections, first, the section-wise in-text citation frequencies were automatically calculated, and then Multiple Regression and Neural Network were applied to the frequencies. The weights produced by Multiple Regression and Neural Network were assigned to sections of articles. The citations existing in specific sections were further multiplied to the section-weights. In the second feature, we calculated the frequency count regardless of their sections. For the third feature, we calculated the similarity of research articles. To classify the citations into important and non-important classes, three machine learning classifiers were used, such as Kernel Logistic Regression, Support Vector Machine and Random Forest. The results revealed that the Neural Network performed better than Multiple Regression. Therefore, Neural Network is better technique to assign the weights. The proposed approach enhanced the value of precision from 0.72 to 0.85.

## Supporting information

S1 File Features of dataset D1. This file contains annotated citation pairs and features of Dataset D1 for analysis.
(CSV)

S2 File Features of dataset D2. This file contains annotated citation pairs and the features of D2 that were utilized for important citation identification.
(CSV)
